# Oral health knowledge, behaviors and parental practices among rural–urban migrant children in Guangzhou: a follow-up study

**DOI:** 10.1186/s12903-017-0385-2

**Published:** 2017-06-07

**Authors:** Ning Pan, Li Cai, Caijuan Xu, Han Guan, Yu Jin

**Affiliations:** Department of Maternal and Child Health, School of Public Health, Sun Yat-Sen University, 74 Zhongshan Road 2, Guangzhou, Guangdong 510080 China

**Keywords:** Migrant children, Oral health, Knowledge, Behaviors, Parental practices

## Abstract

**Background:**

Despite the growing number of rural–urban migrant children in China, follow-up observation on the oral health of migrant children is still scarce. This study described the changes of oral health knowledge, behaviors and parental practices in migrant children over a period of one year. Possible factors affecting changes were also investigated.

**Methods:**

The study used purposive sampling to select five private schools of migrant children in Guangzhou. A total of 1900 students in Grades 3 and 4 were recruited. A self-administered questionnaire was used in November 2011 to understand their basic situations, including oral health knowledge, behaviors and parental practices. A final survey was conducted in April 2013 to detect any changes.

**Results:**

The mean accuracy of oral health knowledge was 53.17% and 59.42% in 2011 and 2013, respectively (*p* < 0.001). For migrant children, the total score of oral hygiene, dietary habits and parental practices increased at the follow-up evaluation (*p* < 0.05). Children with less oral health knowledge were more likely to achieve significantly positive changes in score of knowledge (*p* < 0.001) in the final survey. Migrant children who had worse performance on oral hygiene (beta estimate = 0.68, *p* < 0.001), dietary habits (beta estimate = 0.58, *p* < 0.001) and good parental practices in the baseline survey were more likely to obtain beneficial changes. No significant associations between demographic characteristics and changes of oral health knowledge and behaviors (*p* > 0.05) were observed.

**Conclusion:**

Oral health knowledge, behaviors and parental practices among migrant children significantly improved at the follow-up assessment. However, the overall situation was still poor. Positive and effective health education and prevention programs tailored to rural–urban migrant children with varying levels of oral health knowledge, behaviors and parental practices will be needed.

**Electronic supplementary material:**

The online version of this article (doi:10.1186/s12903-017-0385-2) contains supplementary material, which is available to authorized users.

## Background

With the fast economic development, the internal migrant population in China who generally moved from the countryside to the city has grown rapidly. Guangdong Province has become the largest migrant population province, and has an extensive underage migrant population. Guangzhou is the capital of Guangdong Province, and represents one of the most developed cities in southern China. According to the 2010 data, the total of 2.738 million migrant children under 14 years old in Guangdong Province accounted for 12.0% of the national total, and approximately 20% of the school-age migrant children living in Guangzhou [1]. The government has come to realize the large quantity of migrant children and has embarked on improving their physical and mental health. School-based health education has been implemented, focusing especially on oral health [2].

Dental caries is one of the most common preventable childhood diseases and the most common chronic diseases worldwide, which greatly affects the children’s general health and quality of life [3]. The caries status in China exhibits characteristics typically found in developing countries [4, 5]. Specifically, carious teeth are left untreated among 97% of children aged 5 and 89% of children aged 12 [6].This situation has barely improved, despite the economic development and oral health-care resource provision [7]. In China, rural children always have a lower level of oral health than urban children [8]. The oral health status among rural–urban migrant children may be even worse considering their relatively poor family economic status, less concern from parents, and limited basic health care. By 2011, the overall caries prevalence among rural–urban migrant children in South China was 85.5%, significantly higher than the averages statistics for both rural and urban areas [9].

Adequate oral health knowledge and parental practices including caries attention and diet guidance are essential to equip children with appropriate oral health behaviors [10–13]. A follow-up investigation is necessary and urgent to society’s increased attention to migrant children and the launch of related health planning policies in China [14]. However, information about oral health knowledge, behaviors and parental practices among rural–urban migrant children are still scare. No longitudinal studies on the changes and influencing factors for changes in oral health knowledge and behaviors among migrant children have ever been conducted in China. Therefore, the objectives of the present study were: (1) to describe changes in terms of oral health knowledge, behaviors and parental practices over a period of one year; and (2) to investigate the possible factors influencing the changes of oral health knowledge as well as behaviors, including oral hygiene and dietary habits among migrant children.

## Methods

### Study design

A baseline survey and a follow-up survey were conducted in November 2011 and April 2013 in Guangzhou. We adopted a purposive sampling method, and selected five migrant schools in the Tianhe, Haizhu and Baiyun districts of Guangzhou. This study had obtained permission from headmasters in all participating schools before the survey. Students from all classes of Grades 3 and 4 were recruited from the participating schools as study participants. To ensure that each question was explained similarly to all participants, questionnaires were administered to the whole class during school hours by the same investigator, who were trained and skilled postgraduates. A teacher assisted the administration of the survey to each class. Informed consent forms were sent to the parents of each migrant child prior to the baseline and final surveys, and each parent returned a written consent form.

### Study size

A sample size calculation was established by comparing proportions between the values found in the baseline survey and final survey, using a 90% power (beta) and 5% significance (alpha) for calculation. Based on experience from the previous surveys, migrant children were characterized with high mobility and instability. Thus, we hypothesized that the loss rate was 50%. The sample size was estimated to comprise 1051 migrant children. In total, 1900 students were recruited at baseline and 1081 (56.89%) of them completed the final survey in 2013. The drop-out rate was 43.11%, which was mostly attributed to the high mobility and instability of the migrant population. No differences were found in characteristics (age, sex, family economic status, the baseline scores of oral health knowledge, etc.) between the children who were followed up throughout the study and those who dropped out during the follow-up. A total of 1042 questionnaires remained for analysis, after excluding those ineligible ones.

## Data measurement

### Participant demographics

Basic demographic information included children’s age, grade, gender, number of siblings, age at moving to Guangzhou and family economic status.

### Knowledge of oral health scale

We designed the following 12-item scale to assess migrant children’s oral health knowledge. The items included: (1) Eating sweets in the bedtime can lead to cavities; (2) Frequently eating vegetables can lead to cavities; (3) Failure to brush teeth in the morning and evening can cause tooth decay; (4) Irregular arrangement of teeth can cause tooth decay; (5) Hard toothbrush is harmful to teeth; (6) Cavities should be treated in time; (7) Drinking milk is good for tooth development; (8) Cavities will affect the general health; (9) Toothbrushes should be replaced at least every three months. (10) The most important time to brush teeth is during nighttime; (11) Fluoride toothpaste can prevent cavities; and (12) Failure to drink milk can lead to cavities. Two possible responses, true (counted as 1 score) and false (counted as 0 score), were used to answer the above statements, and the possible total scores ranged from 0 to 12. Higher scores indicated better knowledge regarding oral health (Additional file 1).

### Oral hygiene behavior scale for migrant children

Oral Hygiene Behavior Scale for Migrant Children consisted of five items: (1) “Can each family member have their own toothbrush and cup?” (The possible responses and their corresponding scores: sharing toothbrush and cup, sharing toothbrush, sharing cup and yes = 1, 2, 3, and 4, respectively); (2) “Which manner are you using to brush your teeth every day?” (casual brushing, horizontal brushing, vertical brushing and vertical brushing with horizontal vibration = 1, 2, 3, and 4, respectively); (3) “How long each time do you brush teeth?” (less than 1 min, 1–2 min, 2–3 min, and more than 3 min = 1, 2, 3, and 4, respectively); (4) “Do you rinse your mouth after a meal?”(no = 1,yes = 2); and (5) “How many times do you brush teeth each day?”(none, once, twice and three times or more = 1, 2, 3 and 4, respectively). These behavioral scores were summed, and the total score range was 5–18. Children who obtained higher scores had better oral hygiene behaviors (Additional file 1).

### Dietary habit scale for migrant children

Five items were included in the Dietary Habit Scale for Migrant Children: (1) “How often do you eat chocolate pie or cream cake?”; (2) “How often do you drink carbonated beverages?”; (3) “How often do you eat cheese?”; (4) ”How often do you eat candy?” and (5) “How often do you eat ice cream or butter?” Possible responses included “always, sometimes, or rarely”, and these answers scored “1, 2, or 3”, respectively. The total score range was 5–15. The higher scores indicated better dietary habits (Additional file 1).

### Parental practices scale for migrant children

Six questions regarding parental practices of migrant children, including parental caries attention (two items) and parental diet guidance (four items), were designed by Sun Yat-sen University School of Public Health. The scale of parental caries attention was measured on a 3-point Likert scale with ratings from 1 to 3. The scale of parental diet guidance was measured on a 5-point Likert scale with ratings from 1 (rarely) to 5 (always). The question of “Do your parents often give you money for snacks?” used reverse scoring. Cumulative scores were summed for each scale, with higher scores reflecting more positive parental practices (Additional file 1).

### Statistical analysis

EpiData 3.0 was used for data entry and SPSS 19.0 was used to for data analysis. Descriptive statistics was used to show the participants’ demographics and the chi-square test was applied to evaluate differences according to gender. A paired sample test was performed to analyze the total score and the accuracy of oral health knowledge in rural–urban migrant children. The marginal homogeneity tests compared the changes between the 2011 and 2013 surveys on oral behaviors and parental practices of migrant children. All items were ranked from the most beneficial to the least beneficial change. Ordinal logistic regression assessed the presence and degree of association of independent variables, including participant demographics and the baseline score of oral health knowledge, behaviors and parental practices, with the changes. The changes were grouped into three categories by the difference between the scores of the two surveys. Those with the difference <0, =0, and >0 were classified to have worst changes, no changes and beneficial changes, respectively. A p value <0.05 indicated a statistically significant difference.

## Results

### Demographic distribution of migrant children in five private primary schools

Table 1 shows the demographic information of the 1042 migrant children in the baseline survey. For migrant children, the average age was 9.56 ± 1.00and the gender ratio was 1.33:1 (boys: girls), respectively. The majority had one or more siblings (90.60%). More than half of the migrant children were born in Guangdong Province. Furthermore, 72.08% of the floating children had moved to Guangzhou before they went to the primary school (under six years old), and 65.36% of families were in median or poor economic status. With regard to gender differences, more boys came from other provinces, and girls were more likely to have good living conditions.

**Table 1:** Demographic distribution of migrant children in the baseline survey in 2011

	Total	Boys	Girls	*p-value* ^*a*^
	*n* (%)^b^	*n* (%)^b^	*n* (%)^b^	
Sample size	1042 (100)	594 (57.01)	448 (42.99)	
Age (year), mean ± sd	9.56 ± 1.00	9.67 ± 1.02	9.41 ± 0.94	<0.001
Grade				0.067
3	501 (48.08)	271 (45.62)	230 (51.34)	
4	541 (51.92)	323 (54.38)	218 (48.66)	
Siblings				0.398
0	98 (9.40)	62 (10.44)	36 (8.03)	
1-2	590 (56.62)	330 (55.55)	260 (58.04)	
3 or more	354 (33.98)	202 (34.01)	152 (33.93)	
Place of birth				0.037
Guangdong Province	559 (53.65)	302 (50.84)	257 (57.37)	
Other Province	483 (46.35)	292 (49.16)	191 (42.63)	
Age at moving to Guangzhou				0.055
0 ~ 3	413 (39.64)	228 (38.38)	185 (41.29)	
3 ~ 6	338 (32.44)	183 (30.81)	155 (34.60)	
6 and above	291 (27.92)	183 (30.81)	108 (24.11)	
Family economic status				0.007
Good	361 (34.64)	182 (30.64)	179 (39.96)	
Median	555 (53.26)	335 (56.40)	220 (49.11)	
Poor	126 (12.10)	77 (12.96)	49 (10.93)	

^a^Statistical evaluation using chi-square tests for differences according to gender

^b^All data are expressed as n (%) unless otherwise stated

### Accuracy of oral health knowledge of migrant children in 2011 and 2013

The mean accuracy of oral health knowledge in 2013 in migrant children was 59.42%, which was higher than that in the 2011 survey (53.17%; *p* < 0.001). Table 2 shows a comparison of the accuracy of each item between two surveys, which ranged from 16.22 to 82.05% in 2011 and from 13.05 to 90.79% in 2013. Over a one-year period, the item, “Eating sweets in the bedtimes can lead to cavities,” received the most beneficial change.

**Table 2:** The accuracy of caries-related knowledge of rural–urban migrant children (*n* =1042)

	2011(%)	2013(%)	Change (%)	*p-value* ^*a*^
Eating sweets in the bedtimes can lead to cavities	69.67	89.73	20.06	<0.001
Frequently eating vegetables can lead to cavities	71.59	90.79	19.20	<0.001
Failure to brush teeth in the morning and evening can cause tooth decay	70.44	88.96	18.52	<0.001
Irregular arrangement of teeth can cause tooth decay	77.06	88.58	11.52	<0.001
Hard toothbrush is harmful to teeth	82.05	88.68	6.63	<0.001
Cavities should be treated in time	16.51	22.65	6.14	<0.001
Drinking milk is good for tooth development	16.22	21.31	5.09	0.002
Cavities will affect the general health	75.82	80.52	4.70	0.009
Toothbrushes should be replaced at least every three months	27.74	32.15	4.41	0.019
The most important time to brush teeth is during nighttime	28.02	25.34	−2.68	0.123
Fluoride toothpaste can prevent cavities	78.98	71.50	−7.48	<0.001
Failure to drink milk can lead to cavities	23.61	13.05	−10.56	<0.001

^a^Paired samples test

Only about one-fifth of the sample realized that milk is good for dental health (*n* = 222; 21.31%) and that cavities should be treated in time (*n* = 236; 22.65%); these data were different from the data in the 2011 survey (*n* = 169; 16.22% and *n* = 172; 16.51%, respectively). Approximately, 32% of respondents knew that toothbrushes should be replaced at least every three months, which was also different from the previous survey results (*n* = 289;27.74%). The accuracy of the items “Fluoride toothpaste can prevent cavities” and “Failure to drink milk can lead to cavities” decreased in the 2013 survey (*p* < 0.001). Additionally, no difference in the item “The most important time to brush teeth is during nighttime” was found. Regarding most of the knowledge items, the correct rates in 2013 were significantly higher than those in 2011.

### Oral health behaviors of migrant children in 2011 and 2013

Responses to selected questions about oral health behaviors, including oral hygiene and dietary habits, are shown in Table 3. The percentage of migrant families who had their own toothbrush and cup increased from 56.14 to 67.37% in one year. In the baseline survey, 44.72% of migrant children were found to brush their teeth incorrectly, using methods such as horizontal brushing, vertical brushing, and casual brushing. However, one year later, the proportion dropped to 33.88%. Nearly half of the migrant children (*n* = 513; 49.23%) brushed their teeth for less than two minutes per time in 2011; this number was higher than that in 2013 (*n* = 455; 43.66%). The majority of respondents gargled after a meal (*n* = 403; 41.27%), which was not significantly different from the results of the 2011 survey (*n* = 455; 43.67%). Almost a fifth of the migrant children (*n* = 212; 20.36%) brushed their teeth less than once per day, which differed from the results of the 2011 survey (*n* = 157; 15.07%).

**Table 3:** Oral hygiene and dietary habits among migrant children in 2011 and 2013 (*n* =1042)

	2011 $\left(\overline{x}\pm \mathrm{s}\right)$	2013 $\left(\overline{x}\pm \mathrm{s}\right)$	Change $\left(\overline{x}\pm \mathrm{s}\right)$	*p-value* ^*a*^
Oral hygiene				
Total score	13.98 ± 2.25	14.30 ± 2.19	0.32 ± 2.72	<0.001
Can each family member have their own toothbrush and cup?	3.37 ± 0.87	3.58 ± 0.71	0.21 ± 1.02	<0.001
Yes	56.14%	67.37%		
Sharing cup	31.48%	26.30%		
Sharing toothbrush	5.66%	3.07%		
Sharing toothbrush and cup	6.72%	3.26%		
Which manner are you using to brush your teeth every day?	3.09 ± 1.13	3.29 ± 1.09	0.20 ± 1.42	<0.001
Casual brushing	13.82%	11.90%		
Vertical brushing	12.48%	8.64%		
Horizontal brushing	18.42%	13.34%		
Vertical brushing with horizontal vibration	55.28%	66.12%		
How long each time do you brush your teeth?	2.57 ± 0.97	2.68 ± 0.92	0.11 ± 1.21	0.004
Less than one minute	14.59%	9.88%		
1-2 minutes	34.64%	33.78%		
2-3 minutes	30.23%	35.12%		
More than three minutes	20.54%	21.22%		
Do you rinse your mouth after a meal?	1.56 ± 0.50	1.59 ± 0.49	0.02 ± 0.62	0.213
Yes	56.33%	58.73%		
No	43.67%	41.27%		
How many times do you brush teeth each day?	3.38 ± 0.78	3.17 ± 0.76	−0.21 ± 1.01	<0.001
None	1.63%	0.78%		
Once	13.44%	19.58%		
Twice	29.94%	41.94%		
Three times or more	54.99%	38.00%		
Dietary habits				
Total score	10.33 ± 2.33	10.61 ± 2.15	0.28 ± 2.62	0.001
How often do you eat chocolate pie or cream cake?	1.96 ± 0.74	2.08 ± 0.66	0.12 ± 0.91	<0.001
Always eat	29.27%	18.33%		
Sometimes eat	45.59%	55.66%		
Rarely eat	25.14%	26.01%		
How often do you eat carbonated beverage?	2.00 ± 0.73	2.10 ± 0.65	0.10 ± 0.84	<0.001
Always eat	26.58%	16.31%		
Sometimes eat	47.22%	57.39%		
Rarely eat	26.20%	26.30%		
How often do you eat cheese?	2.19 ± 0.73	2.25 ± 0.72	0.06 ± 0.92	0.026
Always eat	19.19%	16.22%		
Sometimes eat	43.09%	42.71%		
Rarely eat	37.72%	41.07%		
How often do you eat candy?	2.16 ± 0.71	2.20 ± 0.63	0.04 ± 0.84	0.113
Always eat	18.81%	11.90%		
Sometimes eat	46.55%	56.24%		
Rarely eat	34.64%	31.86%		
How often do you eat ice cream or butter?	2.03 ± 0.72	1.98 ± 0.68	−0.05 ± 0.90	0.106
Always eat	24.57%	24.09%		
Sometimes eat	48.18%	53.65%		
Rarely eat	27.25%	22.26%		

^a^Marginal Homogeneity Test

With respect to dietary habits of migrant children, the numbers (percentages) of children who always consumed sweet foods in 2013 were as follows: 191 (18.33%) for chocolate pie or cream cake, 170 (16.31%) for carbonated beverage, 169 (16.22%) for cheese, 124 (11.90%) for candy, and 251 (24.09%) for ice cream or butter. The frequency of eating chocolate pie or cream cake, carbonated beverage and cheese significantly decreased since the 2011 survey (*p* <0.05).

### Parental practices among migrant children in 2011 and 2013

Table 4 reveals responses to questions on parental practices, including attention to children’s caries and diet guidance. Approximately 17.66% of migrant children indicated that their parents rarely paid attention to their teeth, which was different from the 2011 survey (*n* = 271; 26.01%). Additionally, 34.84% of parents dealt with their children’s toothache and caries with a negative attitude. A year later, the proportion was still 30.71%. The mean score of parental attention was 4.67 ± 1.13 and 4.84 ± 1.05, with a significant difference observed between two years (*p* < 0.05).

**Table 4:** Parental practices among migrant children in 2011 and 2013 (*n* =1042)

	2011 $\left(\overline{x}\pm \mathrm{s}\right)$	2013 $\left(\overline{x}\pm \mathrm{s}\right)$	Change $\left(\overline{x}\pm \mathrm{s}\right)$	*p-value* ^*a*^
Parental attention to children’s caries				
Total score	4.67 ± 1.13	4.84 ± 1.05	0.17 ± 1.31	<0.001
Will your parents always pay attention to whether you have caries?	2.14 ± 0.80	2.23 ± 0.73	0.09 ± 0.95	0.002
Always	39.64%	40.69%		
Sometimes	34.35%	41.65%		
Rarely	26.01%	17.66%		
If you have pain with your teeth or have caries, which of the following measures will your parents take?	2.53 ± 0.70	2.61 ± 0.64	0.08 ± 0.86	0.003
Take you to the hospital and follow the doctor’s recommendations	65.16%	69.29%		
Left unchecked, or be ignored	12.09%	8.25%		
Depending on the state	22.75%	22.46%		
Parental diet guidance				
Total score	12.02 ± 3.14	12.29 ± 3.43	0.27 ± 4.01	0.032
Do your parents often give you money for snacks?	3.67 ± 1.10	3.80 ± 1.10	0.13 ± 1.34	0.002
Always	3.74%	4.80%		
Usually	10.65%	7.58%		
Often	27.93%	20.15%		
Sometimes	29.94%	37.43%		
Rarely	27.74%	30.04%		
When your parents give you money, do they tell you what to buy?	2.54 ± 1.38	2.64 ± 1.52	0.10 ± 1.93	0.092
Always	11.42%	18.62%		
Usually	14.78%	13.05%		
Often	23.61%	16.79%		
Sometimes	16.99%	17.08%		
Rarely	33.20%	34.46%		
Do your parents often supervise what kind of snacks you buy?	2.68 ± 1.43	2.71 ± 1.53	0.04 ± 1.91	0.538
Always	15.36%	20.35%		
Usually	15.16%	13.63%		
Often	21.88%	15.16%		
Sometimes	16.99%	18.71%		
Rarely	30.61%	32.15%		
Do you often discuss how to eat is a healthy diet at home with your parents?	3.13 ± 1.36	3.13 ± 1.39	0.00 ± 1.77	0.986
Always	21.02%	22.46%		
Usually	20.44%	20.44%		
Often	25.91%	21.11%		
Sometimes	16.12%	19.87%		
Rarely	16.51%	16.12%		

^a^Marginal Homogeneity Test

No significant changes were observed in parental diet guidance, including informing and supervising what their children buy and eat. Nearly 67.47% of parents sometimes or rarely gave their children money for snacks; this number was higher than that in 2011 (*n* = 601; 57.68%). The mean score of migrant children’s parental diet guidance was 12.02 ± 3.14 and 12.29 ± 3.43, with a significant difference observed between the two years (*p* < 0.05).

### Factors for changes of oral health knowledge, behaviors and dietary habits among migrant children

The results of ordinal logistic regression are presented in Table 5. All models presented a good fit (*p* < 0.001): The parallel line test could not reject the null hypothesis (*p* > 0.05) and the Pearson chi-square goodness-of-fit measure was always non-significant. These summary measures suggested satisfactory ordinal logistic regression models [15].

**Table 5:** Ordinal logistic regression analysis for changes of oral health knowledge, oral hygiene and dietary habits

	Change of oral health knowledge^a^			Change of oral hygiene^b^			Change of dietary habits^c^		
Variables	Regression coefficient	*p-value*	OR (95% CI)	Regression coefficient	*p-value*	OR (95% CI)	Regression coefficient	*p-value*	OR (95% CI)
Intercept_1_ (Beneficial change)	3.93	0.001	.	7.83	<0.001	.	5.37	<0.001	.
Intercept_2_ (No change)	5.04	<0.001	.	8.65	<0.001	.	6.32	<0.001	.
Age (year)	−0.06	0.475	0.94 (0.79,1.12)	−0.05	0.555	0.95 (0.81,1.12)	0.03	0.758	1.03 (0.87,1.21)
Gender									
Boy	0.07	0.610	1.08 (0.81,1.42)	0.09	0.502	1.10 (0.84,1.44)	0.09	0.512	1.09 (0.84,1.42)
Girl	Reference	.	.	Reference	.	.	Reference	.	.
Grade									
3	−0.11	0.518	0.89 (0.64,1.26)	−0.02	0.909	0.98 (0.70,1.36)	0.16	0.341	1.17 (0.85,1.61)
4	Reference	.	.	Reference	.	.	Reference	.	.
Age at moving to Guangzhou									
0 ~ 3	0.17	0.353	1.18 (0.83,1.67)	−0.10	0.546	0.90 (0.65,1.26)	−0.26	0.113	0.77 (0.55,1.06)
3 ~ 6	0.10	0.570	1.11 (0.78,1.58)	−0.01	0.968	0.99 (0.70,1.40)	0.04	0.815	1.04 (0.75,1.45)
6 and above	Reference	.	.	Reference	.	.	Reference	.	.
Siblings									
0	−0.05	0.845	0.95 (0.58,1.56)	−0.30	0.235	0.74 (0.45,1.21)	−0.21	0.382	0.81 (0.50,1.30)
1-2	0.10	0.526	1.10 (0.82,1.47)	−0.02	0.880	0.98 (0.73,1.30)	0.01	0.926	1.01 (0.77,1.34)
3or more	Reference	.	.	Reference	.	.	Reference	.	.
Family economic status									
Good	−0.04	0.862	0.96 (0.60,1.54)	−0.21	0.381	0.81 (0.51,1.29)	0.34	0.145	1.40 (0.89,2.21)
Median	−0.19	0.421	0.83 (0.53,1.31)	0.22	0.342	1.24 (0.80,1.93)	0.12	0.590	1.13 (0.73,1.74)
Poor	Reference	.	.	Reference	.	.	Reference	.	.
Place of birth									
Other Province	0.09	0.537	1.09 (0.83,1.44)	0.15	0.285	1.16 (0.88,1.52)	0.14	0.300	1.15 (0.88,1.49)
Guangdong Province	Reference	.	.	Reference	.	.	Reference	.	.
*Oral health knowledge*									
Baseline score	0.96	<0.001	2.61 (2.33,2.93)	−0.01	0.888	0.99 (0.92,1.07)	−0.02	0.597	0.98 (0.91,1.05)
*Oral health behaviors*									
Baseline score of oral hygiene	−0.06	0.097	0.95 (0.88,1.01)	0.68	<0.001	1.96 (1.81,2.13)	0.02	0.556	1.02 (0.96,1.08)
Baseline score of dietary habits	−0.04	0.189	0.96 (0.91,1.02)	0.05	0.103	1.05 (0.99,1.11)	0.58	<0.001	1.79 (1.66,1.92)
*Parental practices*									
Baseline score of attention to children’s caries	−0.09	0.181	0.92 (0.81,1.04)	−0.19	0.003	0.82 (0.73,0.93)	−0.07	0.264	0.93 (0.83,1.05)
Baseline score of dietary guidance	−0.04	0.090	0.96 (0.92,1.01)	−0.06	0.012	0.95 (0.91,0.99)	−0.06	0.007	0.94 (0.90,0.98)

^a^Model fitting information: chi-square = 445.63(*p* < 0.001); Test of parallel lines: chi-square = 11.81(*p* = 0.694); Goodness-of-fit: (i) Pearson chi-square = 2133.98(*p* = 0.149) and (ii) Deviance chi-square = 1618.62(*p* = 1.00)

^b^Model fitting information: chi-square = 403.54(*p* < 0.001); Test of parallel lines: chi-square = 17.88(*p* = 0.269); Goodness-of-fit: (i) Pearson chi-square = 2007.26(*p* = 0.823) and (ii) Deviance chi-square = 1678.33(*p* = 1.00)

^c^Model fitting information: chi-square = 364.76(*p* < 0.001); Test of parallel lines: chi-square = 11.50(*p* = 0.716); Goodness-of-fit: (i) Pearson chi-square = 2096.30(*p* = 0.321) and (ii) Deviance chi-square = 1780.32(*p* = 1.00)

The children with less oral health knowledge in the baseline were more likely to achieve significantly positive changes in their knowledge score (*p*<0.001) in the final survey. Migrant children who had worse performance on oral hygiene (beta estimate = 0.68, *p* < 0.001) and a higher frequency of consuming sugar-containing food (beta estimate = 0.58, *p* < 0.001) in the baseline survey were more likely to obtain beneficial changes. Regarding parental practices, children whose parents paid more attention to children’s caries (beta estimate = −0.19, *p* = 0.003) and gave children more reasonable dietary guidance (beta estimate = −0.06, *p* = 0.012) were more likely to receive beneficial changes in oral hygiene after one year. Meanwhile, children with parents who gave reasonable dietary guidance were less likely to frequently consume sugar-containing food (beta estimate = −0.06, *p* = 0.007) in 2013. No significant associations between demographic characteristics and changes in oral health knowledge and behaviors (*p* > 0.05) were observed.

## Discussion

Limited data are available to analyze and supervise oral health knowledge, behaviors and parental practices among rural–urban migrant children in China. In general, the situation of oral health knowledge, behaviors and parental practices among migrant children significantly improved during the study, but the overall level was still poor at the follow-up evaluation. Our results showed that oral health knowledge, behaviors and parental practices in the baseline survey were associated with changes of oral health knowledge and behaviors (including oral hygiene and dietary habits) one year later. Migrant children with less oral health knowledge and worse performance on oral behaviors in the baseline were more likely to achieve significantly positive changes. Our findings also confirmed that parental attention to caries and reasonable dietary guidance were related to beneficial changes in the final survey.

The average awareness rate of oral health knowledge was 53.17% among migrant children in the 2011 survey. However, limited studies regarding the level of oral health knowledge of migrant children in China are available. Most previous studies explored the difference between children in urban and rural areas [8, 14]. Compared with previous studies, the level of oral health knowledge among migrant children in Guangzhou is not optimistic. For example, 70.44% children knew that brushing teeth can protect from dental decay while the corresponding percentage in Ethiopia was 85% [16]. Only 27.74% had accurate knowledge on the importance of replacing the toothbrush at least every three months, which was lower than the rate in Nepal [17]. A total of 75.82% of the respondents were aware that dental caries can affect general health, whereas only 16.51% understood that cavities should be treated in time. The level of oral health knowledge increased significantly since the survey in 2011. The reason for the improvement may be related to the existing school-based oral health education programs conducted by the government in Guangzhou, such as teacher training, oral health lectures and knowledge competitions. However, the correct rate of the following two questions, “Fluoride toothpaste can prevent cavities,” and “Failure to drink milk can lead to cavities,” decreased significantly. This finding suggests that certain sorts of knowledge (e.g. the above two) may be less emphasized or even ignored in the current health education programs. A more comprehensive design for health education programs from the government and school is needed to promote the oral health knowledge of migrant children.

Given the lack of oral health knowledge, the majority of migrant children in our study had unhealthy oral behaviors. Approximately 15.07% of migrant children in the baseline survey reported that they brushed their teeth once a day or had never cleaned their teeth over the last month. Nearly half of the respondents brushed their teeth in an incorrect manner and for less than two minutes only. About 43.86% of children shared brushing tools with their family members. These proportions of unhealthy oral behaviors were higher than those found in central and northern China [13, 18] and several other developing countries [17, 19, 20]. The findings may result from poor oral health knowledge among migrant children and insufficient concern from their parents. Over a period of one year, albeit not always statistically significantly, most of oral hygiene behaviors achieved beneficial changes. Migrant children tended to improve the most in their performance on sharing brushing tools with family members, but the least in frequent brushing. Regular and frequent brushing shall be promoted, considering that brushing is the most common and effective method to mechanically remove plaque and white scale [21], and that brushing teeth at least twice a day can more effectively remove plaque and maintain oral hygiene [22]. Overall, oral health education programs should focus on teaching migrant children to brush regularly and correctly and not to share brushing tools with others. Regarding the dietary habits of migrant children, the frequency of consuming sweet-containing food was lower than that of urban children in China [13] and several other developed countries [23]. Moreover, the frequency of consuming chocolate pie or cream cake, carbonated beverage and cheese tended to decrease significantly in the 2013 survey. These findings might be partly explained by the low income among migrant families, who were limited in their financial capabilities to purchase sugar and snacks. To some extent, these situations can help maintain and promote oral health in migrant children.

Previous studies illustrated that the practices of caregivers could influence the health behaviors of their cared [13, 24]. This study showed that the parental practices towards their migrant children, including attention to their children’s caries and dietary guidance, were relatively negative. Only 39.64% of parents were always concerned with the oral health of their children. Nearly a third of parents preferred to ignore children’s toothache and caries development. The dental caries of migrant children did not receive prompt treatment, similar to the situation in most developing countries [17, 25, 26]. This situation had improved in 2013 but was still unsatisfied. These results could be partly explained by the poor economic condition, the lack of medical resources, and the social and cultural isolation of migrant children’s parents. Given the intense life and work pressures, communication between parents and their migrant children is less effective than that of local residents, which reduces the influence of parents on children’s health behaviors [27]. With regard to diet, low income allows only 3.74% of migrant parents to always give their children money for snacks, which to some extent prevents the incidence of unhealthy dietary habits. However, only 11.42% and 15.36% of the parents always supervised the purchase choices of their children. These findings indicated that fewer migrant parents could provide their children with reasonable diet guidance, and such situation did not significantly improve in the finial survey. It is thus recommended that the future health education programs should cover relevant workshops or lectures to improve the migrant parental practices regarding dental health.

Oral health knowledge and behaviors are influenced by many factors. This study shows that the level of oral health knowledge, behaviors and parental practices at the baseline significantly affected the changes of the oral health knowledge, oral hygiene and dietary habits of migrant children in the final survey. The association between oral health knowledge and oral behaviors has also been confirmed in many other studies [28–30]. In the present study, our findings suggest that children with less oral health knowledge were more likely to achieve significantly positive changes one year later. Thus, health education programs should target the different between migrant children with various levels of oral health knowledge, and the dental health curriculum content should be designed depending on the children’s current oral health knowledge. Additionally, we found that migrant children who had a relatively worse performance on oral health behaviors and dietary habits tended to increase their oral hygiene behaviors and to consume sugary food rarely. These findings suggest that school-based dental health programs should pay particular attention to the children with worse oral hygiene and dietary habits, because they are more likely to achieve beneficial changes. Significant associations between the changes of oral behaviors and parental practices were observed; this finding correlates with other studies reporting that parental oral behaviors and attitudes are related with the oral health of children [26, 31, 32]. Migrant children with less parental attention and unreasonable dietary guidance were less likely to achieve beneficial changes in the final survey, indicating that increasing parental practices could positively influence the oral behaviors and dietary habits of migrant children. However, the status quo is that migrant children always experience ineffective communication with parents who are often busy working and confronted with poor living conditions. Migrant children and their parents should both take part in health education programs by strengthening the communication in family members to raise oral health knowledge and promote healthy oral behaviors.

Limitations of this study should be noted. First, five migrant children’s schools were selected by purposive sampling owing to the instability and overcrowding of migrant children. Despite the limitation of sampling, our communication with the local administration and education organizations suggest that the five schools are typical migrant schools in terms of tuition fee, geographic location, and students’ demographic and socioeconomic profiles. Second, the oral health-related behaviors were self-reported, which might be subject to recall bias. Third, a high follow-up loss, which was mostly attributed to the high mobility and instability of the migrant population, was observed. However, no significant differences were found in characteristics (age, sex, family economic status, the baseline scores of oral health knowledge, etc.) between the children who were followed up throughout the study and those who dropped out during the follow-up.

## Conclusion

This study shows that oral health knowledge, behaviors and parental practices among migrant children are significantly improved during a one-year follow-up period; however, the overall situation has remained poor until the end of follow up. The baseline oral health knowledge, behaviors and parental practices is likely to affect the changes of oral health knowledge and behaviors (including oral hygiene and dietary habits). We speculate that the existing school-based health program may be beneficial to the improvement, and thus recommend the government to improve the basic health education system. The future health education system could consider to provide targeted or personalized education programs to the different migrant populations with various levels of oral health knowledge, behaviors and parental practices, and to provide comprehensive oral education programs targeting both children and their caregivers. The oral health of migrants in China requires focused attention and further improvement in the future.

## Additional file

Additional file 1: Questionnaire of Oral Health. We design this questionnaire of oral health to investigate oral health knowledge, behaviors and parental practices among rural–urban migrant children. The same questionnaire was sent to children in the baseline and final survey. (DOC 78 kb)
