# Supplementary material for: Oral health knowledge, behaviors and parental practices among rural–urban migrant children in Guangzhou: a follow-up study
Source: BMC Oral Health. 2017 Jun 7;17:97. doi: 10.1186/s12903-017-0385-2 (PMC5461744; doi:10.1186/s12903-017-0385-2)
Supplement: Additional file 1: — Questionnaire of Oral Health. We design this questionnaire of oral health to investigate oral health knowledge, behaviors and parental practices among rural–urban migrant children. The same questionnaire was sent to children in the baseline and final survey. (DOC 78 kb) [file 12903_2017_385_MOESM1_ESM.doc]

**Questionnaire of Oral Health**

**Dear students：**

Hello!

In order to investigate oral health knowledge and behaviors of pupils, we have designed this questionnaire. The information that you provide will help us to develop health education measures. We will keep the answers confidential. Please answer the following questions truthfully and fill "✓" in front of your choice. Thank you very much for your cooperation.

**Sun Yat-Sen University**

**School：**__________ **Grade：**________ **Class：**________ **Sex：**□Boy □Girl

**Name：**___________ **Birthday：**____ **Place of birth：**_________

**How many siblings do you have：** □0 □1~2 □3 or more

**Age at moving to Guangzhou：** □0~3 □3~6 □6 and above

**How is your family's economic status：**□Good □Median □Poor

- **Part 1**

| ***Knowledge of Oral Health Scale*** | |
| --- | --- |
| **1. Eating sweets in the bedtime can lead to cavities.** | □True □False |
| **2. Frequently eating vegetables can lead to cavities.** | □True □False |
| **3. Failure to brush teeth in the morning and evening can cause tooth decay.** | □True □False |
| **4. Irregular arrangement of teeth can cause tooth decay.** | □True □False |
| **5. Hard toothbrush is harmful to teeth.** | □True □False |
| **6. Cavities should be treated in time.** | □True □False |
| **7. Drinking milk is good for tooth development.** | □True □False |
| **8. Cavities will affect the general health.** | □True □False |
| **9. Toothbrushes should be replaced at least every three months.** | □True □False |
| **10. The most important time to brush teeth is during nighttime.** | □True □False |
| **11. Fluoride toothpaste can prevent cavities.** | □True □False |
| **12. Failure to drink milk can lead to cavities.** | □True □False |

- **Part 2**

| ***Oral Hygiene Behavior Scale for Migrant Children*** |
| --- |
| **1. Can each family member have their own toothbrush and cup?** |
| □Sharing toothbrush and cup □Sharing toothbrush □Sharing cup □Yes |
| **2. Which manner are you using to brush your teeth every day?** |
| □Casual brushing □Horizontal brushing □Vertical brushing  □Vertical brushing with horizontal vibration |
| **3. How long each time do you brush teeth?** |
| □Less than 1 min □1-2 min □2-3 min □More than 3 min |
| **4. Do you rinse your mouth after a meal?** |
| □Yes □No |
| **5. How many times do you brush teeth each day?** |
| □None □Once □Twice □Three times or more |

- **Part 3**

| ***Dietary Habit Scale for Migrant Children*** | |
| --- | --- |
| **1. How often do you eat chocolate pie or cream cake？** | □Always □Sometimes □Rarely |
| **2. How often do you drink carbonated beverages？** | □Always □Sometimes □Rarely |
| **3. How often do you eat cheese？** | □Always □Sometimes □Rarely |
| **4. How often do you eat candy？** | □Always □Sometimes □Rarely |
| **5. How often do you eat ice cream or butter?** | □Always □Sometimes □Rarely |

- **Part 4**

| ***Parental Practices Scale for Migrant Children*** |
| --- |
| - **Parental attention to children’s caries** |
| **1. Will your parents always pay attention to whether you have caries?** |
| □Always □Sometimes □Rarely |
| **2. If you have pain with your teeth or have caries, which of the following measures will your parents take?** |
| □Take you to the hospital and follow the doctor’s recommendations  □Left unchecked, or to be ignored  □Depending on the state |
| - **Parental diet guidance** |
| **1. Do your parents often give you money for snacks?** |
| □Always □Usually □Often □Sometimes □Rarely |
| **2. When your parents give you money, do they tell you what to buy?** |
| □Always □Usually □Often □Sometimes □Rarely |
| **3. Do your parents often supervise what kind of snacks you buy?** |
| □Always □Usually □Often □Sometimes □Rarely |
| **4. Do you often discuss how to eat is a healthy diet at home with your parents?** |
| □Always □Usually □Often □Sometimes □Rarely |

**Thank you very much for your patience and sincere cooperation!**
